# Exploring the Impact of Structural Modifications of Phenothiazine-Based Novel Compounds for Organic Solar Cells: DFT Investigations

**DOI:** 10.3390/polym17010115

**Published:** 2025-01-05

**Authors:** Walid Taouali, Amel Azazi, Rym Hassani, Entesar H. EL-Araby, Kamel Alimi

**Affiliations:** 1 Research Laboratory of Asymmetric Synthesis and Molecular Engineering of Materials for Organic Electronic (LR18ES19), Department of Physics, Faculty of Sciences of Monastir, University of Monastir, Avenue of Environment, Monastir 5019, Tunisia; kamel.alimi@fsm.rnu.tn; 2 Department of Physical Sciences, Physics Division, College of Science, Jazan University, P.O. Box 114, Jazan 45142, Saudi Arabia; eelaraby@jazanu.edu.sa; 3 Environment and Nature Research Centre, Jazan University, P.O. Box 114, Jazan 45142, Saudi Arabia; rhassani@jazanu.edu.sa

**Keywords:** density functional theory, reduced density gradient, open-circuit voltage, charge transfer length, transition density matrix

## Abstract

This paper explores a novel group of D-π-A configurations that has been specifically created for organic solar cell applications. In these material compounds, the phenothiazine, the furan, and two derivatives of the thienyl-fused IC group act as the donor, the π-conjugated spacer, and the end-group acceptors, respectively. We assess the impact of substituents by introducing bromine atoms at two potential substitution sites on each end-group acceptor (EG1 and EG2). With the donor and π-bridge held constant, we have employed density functional theory and time-dependent DFT simulations to explore the photophysical and optoelectronic properties of tailored compounds (M1–M6). We have demonstrated how structural modifications influence the optoelectronic properties of materials for organic solar cells. Moreover, all proposed compounds exhibit a greater V_oc_ exceeding 1.5 V, a suitable HOMO-LUMO energy gap (2.14–2.30 eV), and higher dipole moments (9.23–10.90 D). Various decisive key factors that are crucial for exploring the properties of tailored compounds—frontier molecular orbitals, transition density matrix, electrostatic potential, open-circuit voltage, maximum absorption, reduced density gradient, and charge transfer length (D_index_)—were also explored. Our analysis delivers profound insights into the design principles of optimizing the performance of organic solar cell applications based on halogenated material compounds.

## 1. Introduction

Over recent years, organic photovoltaic (OPV) devices have attracted a lot of attention because of their promise to provide lightweight, flexible, and semitransparent nature, and inexpensive solar energy solutions [1,2,3]. The development in molecular design strategies and efficiency enhancement in OPVs have been thoroughly collected in recent works [4,5,6,7], which offer a detailed background for comprehending updates of the field and highlight important limits that still need to be addressed. Non-fullerene acceptors (NFAs) progress has drawn considerable interest in the sector of organic photovoltaic applications in light of their advanced properties exceeding the limitations of traditional fullerene acceptor material [8,9,10,11]. The donor–spacer–acceptor (D-π-A) structure has attracted a lot of interest among different molecular configurations because it can improve light absorption, speed up charge transfer procedures, and adjust electrical characteristics. Within the diverse NFAs, end-groups (EGs) of the fused-ring electron acceptors (FREAs) are the source of improving the overall performance of these materials [12,13,14]. Their tunable planarity and strong electron-withdrawing ability provide a major influence on the optical and electronic properties of NFAs [15,16]. These properties allow for fine-tuning of charge transfer properties and absorption spectra and facilitate intermolecular π-π stacking. Consequently, understanding the effect of EG modifications on the photo-physical and opto-electronic properties of FREAs is crucial for the rational design of high-performance NFAs. Several types of innovative building materials, including cyanide (CN), 1-dicyanomethylene-3-indanone (IC), malononitrile, diketopyrrolopyrrole (DPP), and rhodamine, have been exploited as EGs for FREAs [17,18,19]. The most efficient end-group in these compounds is the IC group and its derivatives. Since the discovery of the IC group, more than fifty IC derivatives designed to enhance the performance properties of NFAs [20,21].

Among the derivatives of IC groups, thienyl-fused IC group compounds are especially notable due to their wide-ranging applications in organic electronics [22,23,24]. The optoelectronic properties of FREAs can be affected by thienyl positions. Studying substituent effects on molecular systems plays a decisive role in modulating and predicting their chemical properties and reactivity. The introduction of halogens such as chlorine and bromine into these systems can alter their electronic distribution, intermolecular interactions, and overall stability, consequently influencing their optoelectronic properties.

On the other hand, phenothiazine (PTZ) and the materials derived from it have gained popularity in a number of optoelectronic applications in recent years, due to their distinct optical and electrical properties, flexibility in functionalization, affordability, and commercial availability [25,26,27,28]. Moreover, it was demonstrated by the evaluation of organic photovoltaic performance that adding a spacer does not always result in higher power conversion efficiency. However, it was found that polymers possessing a furan π-spacer were somewhat more effective than their thiophene counterparts and more potent than those without a π-spacer [29,30,31].

**Figure 1:**
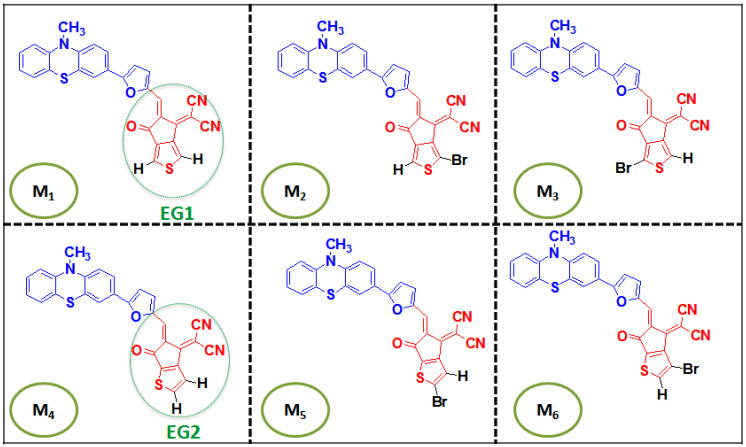
Two-dimensional molecular geometries of all tailored compounds.

Hence, in this study, phenothiazine as the donor material (D) bridged with furan π-spacer to thienyl-fused IC end-groups (A) was used to give D-π-A type of chromophores configuration as mentioned in Figure 1. It is expected that the suggested compound will have good solubility in common organic solvents, including toluene, chloromethane, and chloroform, based on the structural properties of the designed molecules and similar systems documented in the literature [32,33,34,35]. The aromatic framework, combined with heteroatoms like sulfur and oxygen in the fused system, enhances its compatibility with these solvents. Furthermore, methods like adding alkyl groups are well-established for enhancing solubility even more, when necessary, without affecting the electronic properties of these compounds [36,37]. In this perspective, we have highlighted how structural change, such as bromine atom substituents and the change inthienyl positions of the fused-ring electron acceptors, influences the optoelectronic properties. This work notably explores the influence of bromine (Br) substitution on two thienyl-fused (IC) derivatives denoted EG1 and EG2. EG1 and EG2 contain identical atoms but differ in the positions of their thienyl group. Each molecule of them presents two potential substitution sites 1 and 2 (see Supplementary Figure S1).With the substitution of bromine (Br) atoms at these sites, our goal is to clarify how structural modifications influence the optoelectronic properties of the D-π-A configurations. Our results will offer deeper understanding into the design strategies for enhancing the performance of novel compounds in advanced organic electronic applications.

## 2. Computational Methodology

Gaussian 09 software [38] was used to carry out calculation approaches employing density functional theory (DFT) and time-dependent density functional theory (TD-DFT). The optimization of molecular geometries was performed through the B3LYP/6-311G(d,p) level of theory [39,40], focusing on studying energies and electronic structures of proposed molecules M1–M6. Electronic excitations and absorption spectra were assessed through the TD-DFT B3LYP/6-311G(d,p) level of theory [41]. B3LYP functional was chosen for the theoretical simulations due to its demonstrated accuracy in predicting the optoelectronic characteristics and geometries of various organic compound. Properties of charge transport and distribution of all substituted molecules were simulated according to results derived from DFT simulations. Fundamental parameters derived from DFT simulations, including electron localization function (ELF) maps, transition density matrix, molecular orbitals, electronic transitions, ∆r index, D index, and dipole moment, were carefully analyzed for the tailored molecules, and examined and presented using the software packages Gauss View 5 [42], Swizard [43], VMD 1.9 [44],and Multiwfn [45].

## 3. Results

### 3.1. Geometry Optimization

We created six new structures in our study by changing the terminal acceptors labeled as M1 to M6, which are divided into two distinct compounds based on the position of the sulfur (S) atom. Compound group 1 includes M1, M2, and M3, while compound group 2 consists of M4, M5, and M6. However, within each group of compounds, every structure under investigation has a unique terminal acceptor that differs by the position of the bromine (Br) substituent. All tailored compounds share the identical donor phenothiazine (PTZ) linked with the furan π-spacer.

In order to carry out structural optimization of the recently tailored compounds, the functional B3LYP was utilized with basis set 6-311G(d,p). To proceed with additional computations in subsequent molecular simulations, it is imperative to optimize the molecular configurations. Figure 1 shows the graphic representation of every derived structure from unsubstituted structures (M1 and M4). The scrutinized compounds are divided into two fragments: a newly substituted acceptor (red) characterized by low electron density and a donor (blue) with high electron density.

**Figure 2:**
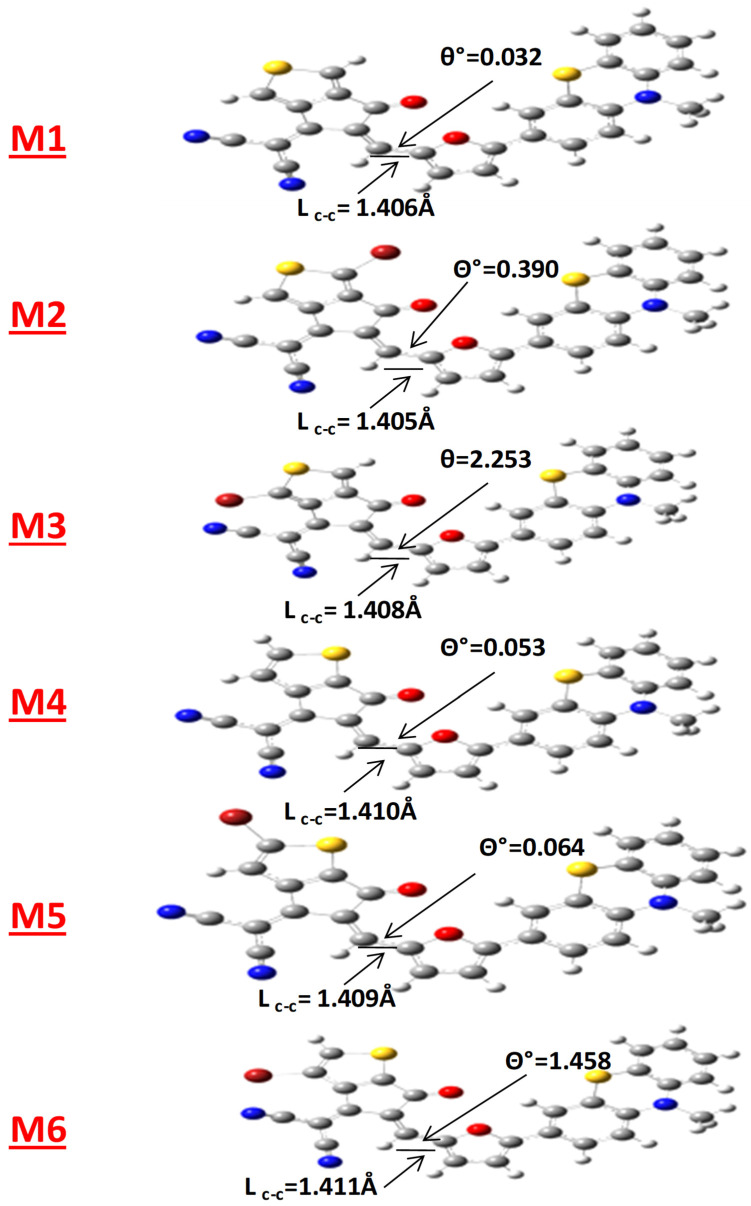
The optimized configurations of tailored compounds illustrating bond lengths (L_c-c_) and dihedral angle (θ) between the π-spacer and the end-group acceptor.

The ground state structures of the molecules have a close relationship with the optoelectronic properties. The bond lengths (L_c-c_) and dihedral angle (θ) of designed structures were determined using geometry optimizations. These parameters are shown in Figure 2. Table 1 tabulates these computationally produced results of investigated parameters. The bond length between the donor and acceptor of designed structures fluctuates between 1.405 and 1.411 Å, falling between the L_c-c_ of double bond C=C (1.34 Å) and the L_c-c_ of carbon–carbon single bond (1.54 Å). This demonstrates considerable π-electron conjugation between the donor unit and the terminal acceptors [46,47]. The degree of conjugation determines the structural characteristics of all compounds.

Computationally created molecules have dihedral angles that range from 0.032° to 2.253°. This reveals molecular planarity, leading to increased resonance and faster charge transfer. The donor and acceptor can rotate freely with less obstruction and prominent planarity when the dihedral angle (θ) is minimal. Planarity is represented by both of these parameters (L_c-c_ and θ), and strong conjugation may enhance the optoelectronic properties of these compounds.

The planarity of the structures under study was evaluated by looking at the molecular planarity parameter (MPP) and the span of deviation from the plane (SDP) utilizing Multiwfn 3.8 and VMD 1.9.3 [48]. As seen in Table 1, the molecular coplanar behavior is indicated by the MPP, although the degree of molecular component divergence from the fitted plane is revealed by the SDP. Planarity and MPP exhibit an inverse correlation. The MPP values for investigated compounds (M1 to M6) range between 0.433 and 0.777, indicating a moderate to high degree of planarity. It is clear that M1 and M4, which lack of bromination, exhibit higher planarity compared to brominated molecules. Consequently, bromination at specific sites, as observed in M2, M3, M4, and M5, may generate steric hindrances that marginally reduce the planarity.

Moreover, the SDP values of generated structures are ranging from 1.970 to 2.110 Å, and the lowest SDP values are attributed to the compounds without bromine atoms (M1 and M4), which predict the critical coherence between the SDP and MPP values.

### 3.2. Frontier Molecular Orbitals

**Figure 3:**
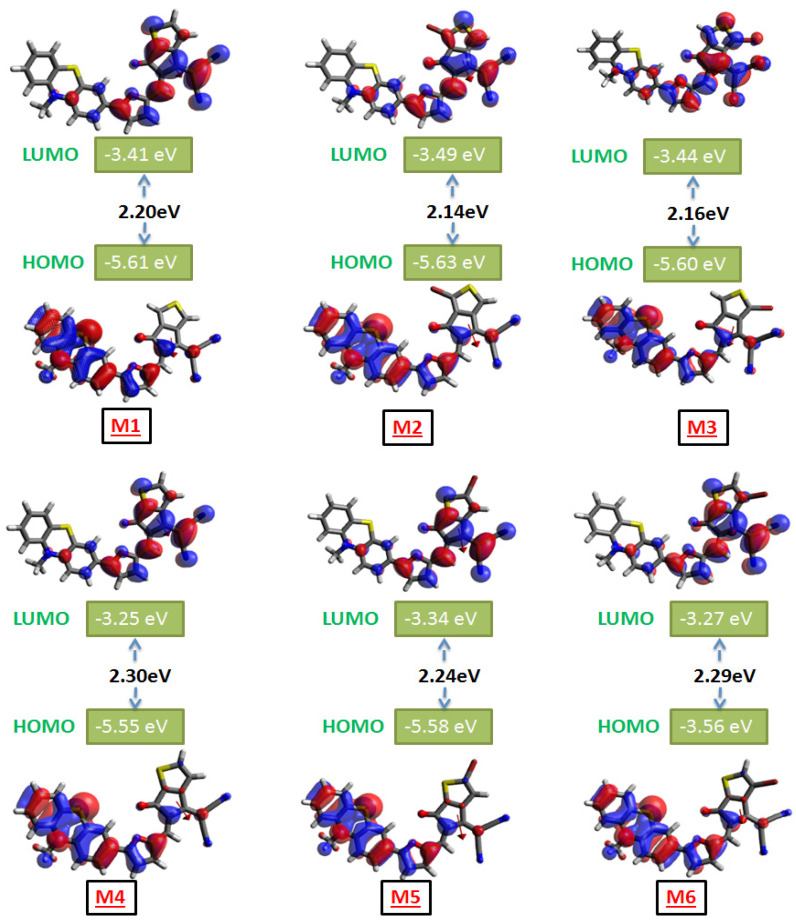
FMOs of all studied compounds associated with their HOMO-LUMO energy gap.

The energy levels of molecular orbitals have a great impact on organic solar cells (OSCs) because they affect the way charges are transported from the donor unit to the acceptor unit. The redistribution of charge density between the ground and excited state energy levels is represented by frontier molecular orbitals (FMOs). When an electron is excited, it effectively moves from the highest occupied molecular orbital (HOMO) to the lowest unoccupied molecular orbital (LUMO) state. Figure 3 demonstrates that the LUMO energy level is located in the acceptor fragment of the studied compounds and the HOMO energy level is located in the donor fragment. The HOMO-LUMO energy difference presents a crucial parameter, which controls the material’s ability to absorb light and exerts an effect on charge mobility as well as solar cell efficiency. Supplementary Table S1 demonstrates how variations in the positions of bromine (Br) atoms directly affect the dipole moment of the end-group, consequently affecting the LUMO electron distribution of the non-fullerene acceptor (NFA). Moreover, the electron localization function (ELF) maps furnish a detailed visualization of electron density distribution and localization within each atom of the end-group molecule. It is widely employed in computational molecular science to provide additional information beyond conventional bonding descriptors derived from electron density [49,50,51]. As seen in Supplementary Figure S2, the color red signifies increased electron density localization, whereas blue color indicates a decreased concentration of localized electron density. On the ELF map, the bromine atom in tailored compounds is surrounded by a noticeable blue ring, indicating a considerable degree of electron delocalization. The electron cloud arrangement of adjacent atoms in substituted structures at position 1 presented in 1Br-EG1 and 1Br-EG2 structures closely resembles that observed in molecules EG1 and EG2. However, there is a slight difference observed in the malonitrile region of molecules EG1 and EG2 when the brome was substituted at position 2. Thus, our design strategy begins with optimizing the dipole moment and elevating the LUMO energy levels of the end-groups, which effectively suppresses the V_oc_ loss in organic solar cells (OSCs). This approach serves as an initial hypothesis for enhancing the photovoltaic properties of NFAs.

The FMOs of the developed compounds are shown in Figure 3, with colors according to the strength of charge distribution. Areas of high potential are indicated by red colors, whereas low-potential orbitals are indicated by blue colors. Higher solar cell efficiency and quick excitation are generally linked to a LUMO positioned at a low energy level and a HOMO found at a high energy level. As seen in Figure 3, the energy levels of the HOMO are essentially located in the donor areas of all tailored structures. The electron density of the LUMO is found on both the acceptor and donor units. However, in contrast to the HOMO, the LUMO electron density of all designed compounds was redistributed toward the end-group acceptors. All studied compounds exhibit a charge shift from donor fragment to end-group acceptor areas, indicating the occurrence of intermolecular charge transfer. This property enables these materials to function as effective OSCs. The arrangement of the HOMO-LUMO gap values across all tailored compounds ranks in the following orders: M4 (2.30 eV) > M6 (2.29 eV) > M5 (2.24 eV) and M1 (2.20 eV) > M3 (2.16 eV) > M2 (2.14 eV). These results predict that all designed compounds possessed narrower HOMO-LUMO energy gaps around 2.2 eV. Organic materials used in optoelectronic devices, such as organic solar cells, typically include these energy gaps; a smaller gap can improve light absorption and charge transport. The change in the energy gap shows how structural elements like sulfur positioning and bromination affect the electronic properties of the compound. The position of the sulfur atom significantly affects the energy gap, leading to a reduction of approximately 0.1 eV depending on its location. A notable difference of 0.16 eV in the HOMO-LUMO energy gap can be observed with Br substitution along with the modification of the sulfur atom position.

Moreover, Koopman’s theorem states that overall reactivity features of the studied compounds are primarily analyzed in order to determine their stability and reactivity. The parameters that are described using the energy gap E_g_, include the ionization potential (IP = −E_HOMO_), electron affinity (EA = −E_LUMO_), chemical potential (μ), electrophilicity index (ω), and global hardness (η) [52,53,54]. Equations (1)–(3) are used to calculate the above mentioned parameters.

(1) $$\mu =-X=\frac{E_{CV\;HOMO}-E_{CV\;LUMO}}{2}$$

(2) $$\eta =E_{CV\;LUMO}-E_{CV\;HOMO}$$

(3) $$\omega =\frac{\mu^{2}}{2\eta }$$

The reactivity parameter values are tabulated in Table 2. It is clear that the substitution of bromine in position 1 leads M2 and M5 to have the largest negative chemical potential (μ) values, at −4.56 eV and −4.46 eV, respectively, indicating their highest stability. Moreover, these structures M2 and M5 have the lowest values of hardness, 1.07 eV and 1.12 eV, respectively, relative to their primary compounds M1 and M4 (1.10 eV and 1.15 eV, respectively), which indicates that these compounds do not resist electron transfer. A molecule’s electrophilicity value reveals its ability to draw electrons and function as an electrophile, where a lower number denotes its ability to donate electrons and function as a nucleophile. It is remarkable that all of the substituted structures have more electrophilicity than their primary structures due to the substituent of bromine. Moreover, one of the most intriguing characteristics of OSCs is drawn in the higher value of dipole moment (ρ), which enhances the stability of these compounds and results in superior charge transfer CT. All tailored compounds exhibited a higher value of dipole moment exceeding 9 Debye, which confirm their stability. It is clear from this table that halogenated structures possess greater dipole moments than unsubstituted structures.

### 3.3. Analysis of Electrostatic Potential (MEP)

**Figure 4:**
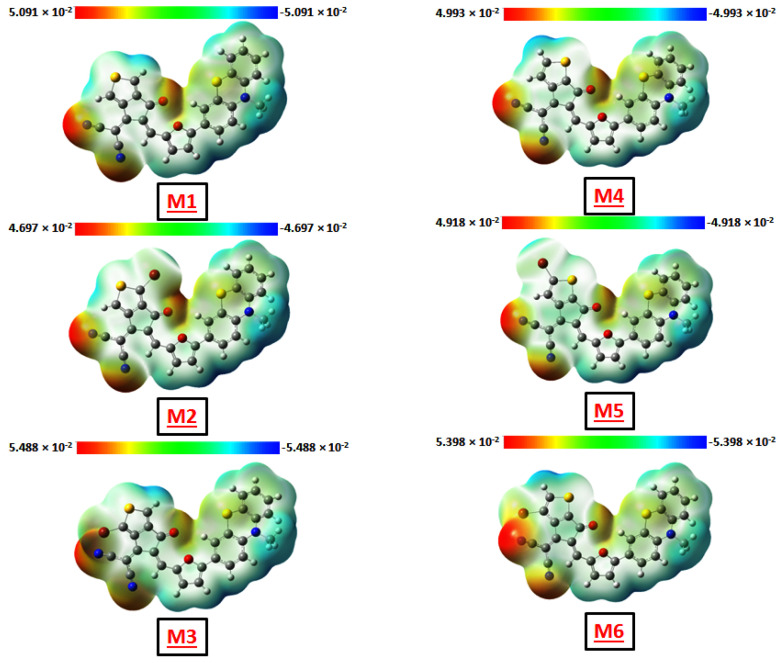
Molecular electrostatic potential of all designed compounds (M1–M6).

The molecular electrostatic potential (MEP) surface has been created to estimate both nucleophilic and electrophilic areas of chemical attack on the designed compounds. The interaction zones can be found on these sites [55,56]. As seen in Figure 4, the nucleophilic reactive attack sites are indicated by blue dots with positive electrostatic potential values, while electrophilic reactive attack sites are indicated by red dots with negative electrostatic potential values. Green dots represent the neutral zone of electrostatic potential. Figure 4 clearly shows the total electron density on which the MEP mapping was performed of all designed compounds. It also demonstrates the relative reactivity of the atoms and chemically active places. At every structure under investigation, the atoms with the highest electron densities are the nitrogen and oxygen ones at the terminal acceptor groups. Given the large charge dispersion with separate zones of all tailored compounds, it can be assumed that they are all great candidates for high-efficiency organic solar cells.

### 3.4. Reduced Density Gradient

**Figure 5:**
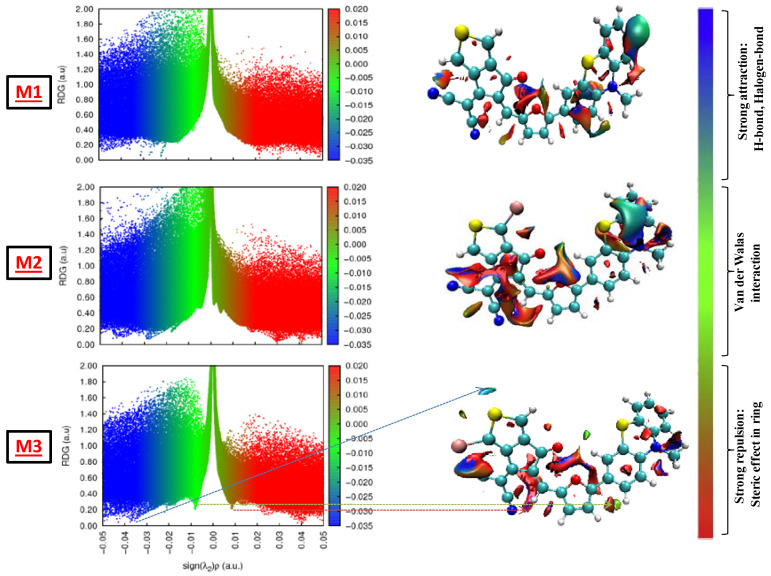

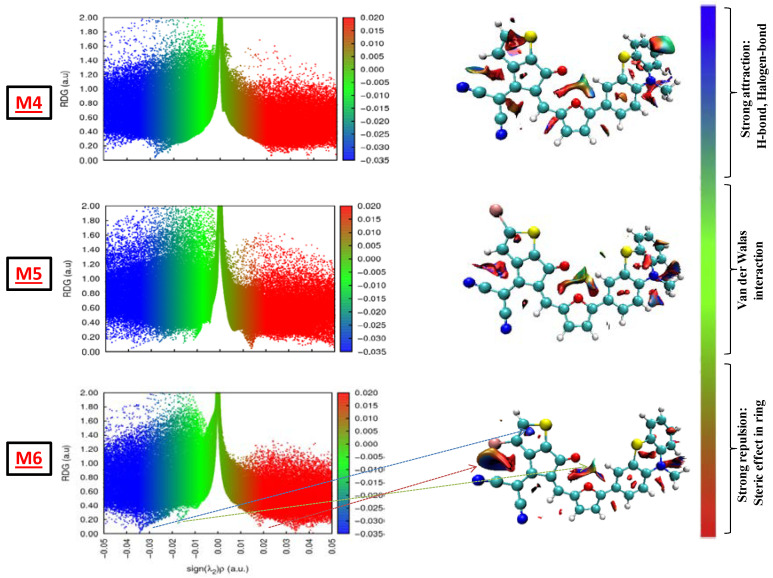
NCI-RDG and interaction types of all tailored compounds.

Reduced density gradient (RDG) calculations based on the examination of non-covalent interactions (NCI) can be used to identify weak intramolecular or intermolecular interactions found in our study [57,58,59]. These interactions can contribute to the stability of the molecular structure. The NCI-RDG methods for all studied compounds were carried through an isosurface value of 0.3, and their results are displayed in Figure 5. The RDG isosurface around their real space can be used to identify the different types of interaction; the blue, green, and red regions, respectively, represent the steric effect, vdW interactions, and H-bond interactions. The strongest non-covalent interactions are represented by the peaks. All of the graphs demonstrate that van der Waals and attractive forces predominate steric force, indicating that all of these tailored compounds are stable, precisely structures substituted by bromine in position 2.

### 3.5. Optical Properties

**Figure 6:**
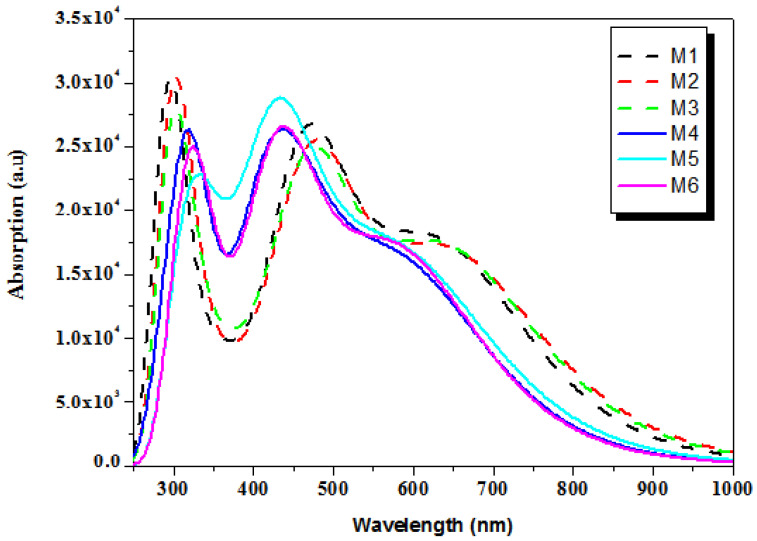
Absorption spectra of all proposed compounds (M1–M6).

Modification of the optical and electrical characteristics of polymers can either greatly accelerate or profoundly slow down their photovoltaic properties. Lower excitation energy, increased oscillator strength, and a wide absorption spectrum are necessary to create an OSC with exceptional performance. All of the designed structures showed an impressive ability to absorb energy in the visible and near-IR regions, which is stated from 400 nm to nearly 950 nm, as shown by the graph in Figure 6. Table 3 provides an overview of the studied compounds, as well as the excitation energy, oscillator strength, maximum absorption, and intramolecular charge transfer contribution. The computed results indicate that the main peak corresponds to the singlet HOMO→LUMO transition with a contribution exceeding 90%. It is observed that the substitution of compounds M1 and M3 with bromine atoms does not have a significant effect on the oscillator strength values f. The simulated λ_max_ values of proposed compounds are increasing in the following trends: M4 (620.47 nm) < M6 (626.49 nm) < M5 (639.12 nm) and M1 (638.10 nm) < M3 (648.88 nm) < M2 (654.02). It is evident that all of the substituted structures with bromine substituent atoms enhance their primary compound absorptions (M1 and M4), which indicates improved light absorption. Since M2 has the smallest HOMO-LUMO energy gap (2.14 eV), it showed the highest λ_max_ value, which is advantageous for enhancing solar energy conversion efficiency.

Determining the excited state characteristic is crucial to understanding the electrical configuration and properties of these compounds. Numerous indices such as D_index_, S_r_, t_index_, and ∆r have been calculated to depict the first excited state.

The distance between the hole centroid and the electron centroid is known as the D_index_. ∆r index of these compounds was computed at the same theoretical level, which may be used to evaluate the average hole–electron distance upon photo-excitation, in order to verify the accuracy of the computed D_index_. The ∆r index provides a quantitative assessment for accessing the charge transfer length (D_index_) of electron excitation; a larger ∆r index correlates with a longer CT distance. The overlap distance between the centroid of the hole and the electron is expressed by the overlap integral Sr. Efficient exciton dissociation enhances charge transfer in the photo-electric conversion process of organic solar cells. For the purpose of comparing the exciton separation ability of studied compounds, the key parameters of electron excitation of the first five excitations were computed and tabulated in Table 4. From this table, the ∆r indices of all proposed compounds suggest that the excitations from ground state (S_0_) to excited state (S_1_) acquire strong charge transfer excitations because their ∆r indices are sufficiently large (2.0 Å serves as a criterion for discerning between local excitation (LE) and charge transfer excitations (CT) [60,61]). After looking at the Sr index, it was found that substituted compounds with bromine have smaller Sr indices than their primary structures, mostly because of their elevated D_index_ value. In particular, M6 possesses a minimal Sr value of 0.321, which suggests that there may be an overlap between the hole and electron in the first excited state. The t_index_ values of all studied compounds exhibit positive values, indicating that in their first excited state, holes, and electrons are significantly separated from one another [62].

### 3.6. Transition Density Matrix

**Figure 7:**
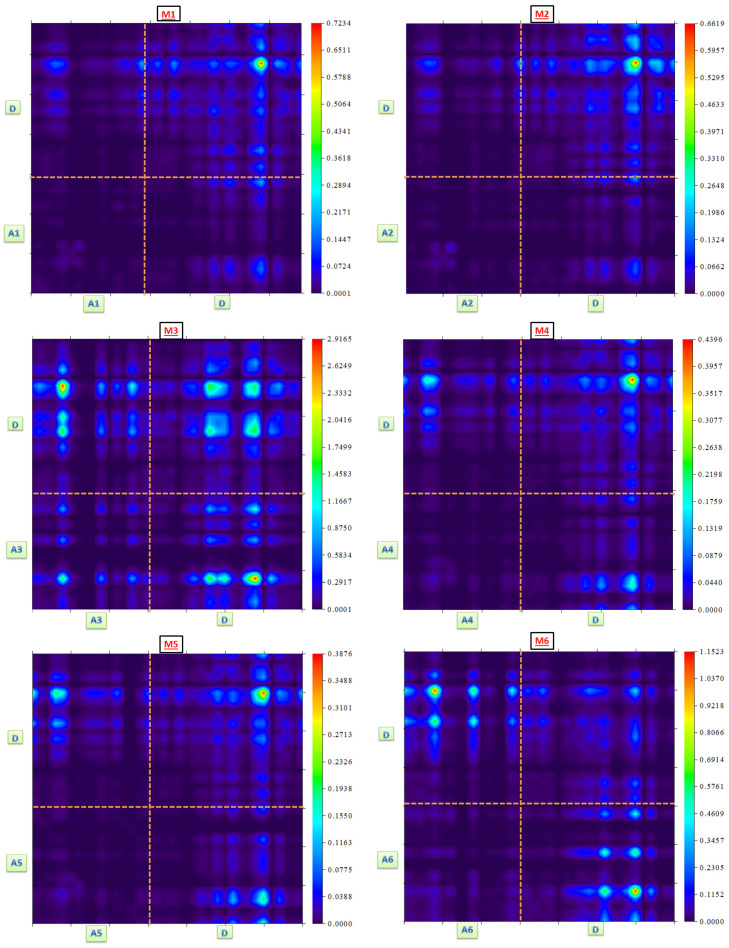
TDM plots showing electron density distribution across A–D fragments of all compounds. (D: Donor + π spacer; A: end-group acceptor).

The electronic excitation, acceptor-donor interactions, and electron–hole localization are all examined by the transition density matrix (TDM) [63,64]. The electron coherence and donor connection of each structure are most clearly depicted in TDM maps. Because of their minimal impact on significant transitions, the hydrogen atoms are excluded from our analysis. The identification of electron–hole pair delocalization is aided by the first excited state S_1_. Each compound is separated into donor and acceptor parts as shown in Figure 1. As shown in Figure 7, TDM maps for every tailored compound are obtained using Multiwfn 3.8, which is useful in some of the previously described parameters. A closer examination reveals that the acceptor’s area of M3 and M6 compounds had more brilliant fringes. Fringes in diagonal positions manifest the extent of localization of electron density, while those in off-diagonal positions refer to the charge transfer process in these structures. Moreover, it is observed that the acceptor portions of these two structures exhibit noticeably reduced dark spot areas, suggesting that in the aforementioned compounds, the ratio of excited electrons that moved from the donor to the acceptor region is quite high. The hole–electron coherence in the tailored compounds obeys the subsequent order M3 (2.91) > M1 (0.72) > M2 (0.66) > M6 (1.15) > M4 (0.44) > M5 (0.39), which is determined by the coefficient interactions between the donor and acceptor portions. This interaction order shows that M3 exhibits a weak hole–electron coupling, which results in an efficient exciton dissociation. These results support the finding of higher D_index_ and ∆r values obtained for compound M3.

### 3.7. Exciton Binding

An OSC device’s productivity is regulated by exciton binding energy (E_b_), which is the minimal amount of energy required for exciton dissociation. Electrical energy is produced when specific radiation is absorbed because the excitons become separated and are drawn to the electrodes in which they are located. The enhanced charge separation is stimulated by the lowest E_b_, which increases charge mobility. The next equation is utilized to calculate the E_b_ values of all tailored compounds [65]:

E_b_(M) = E_g_(M) − E_opt_(M) (4)

The energy of the HOMO-LUMO gap is represented by the term E_g_(M), and E_opt_ represents the excitation energy for the first state S1. Table 5 summarizes the quantitative results of all designed compounds. The ranking of E_b_ for all designed compounds is listed as follows: M6 (0.31 eV) > M6 = M5 (0.30 eV) > M1(0.26 eV) > M3 (0.25 eV) > M2 (0.24 eV). According to the binding energy results, M3 exhibits the lowest value, indicating higher and easier electron–hole pair dissociation in the excited state.

### 3.8. Open-Circuit Voltage (V_oc_) and Fill Factor

**Figure 8:**
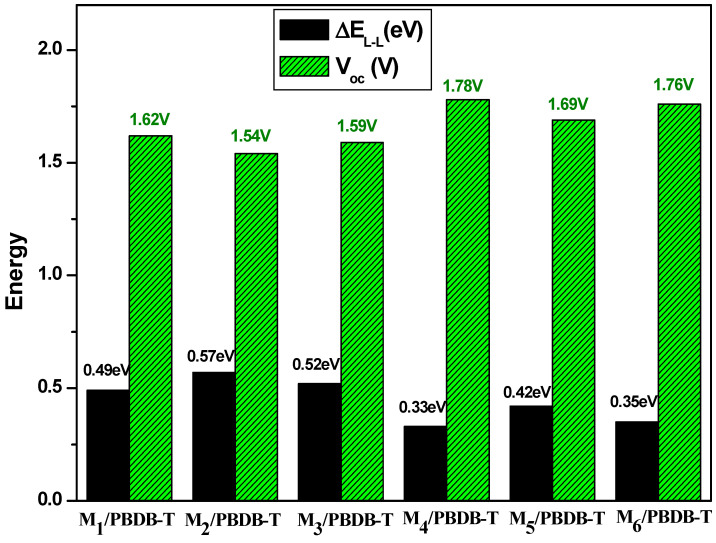
Open-circuit voltage (V_oc_) and LUMO _offset_ (∆E_L-L_)for all studied molecules.

The open-circuit voltage (V_oc_) parameter is significant in organic solar cells since it is used to measure the working capabilities and performance of OSCs. If the HOMO value of the donor is lower and the LUMO value of the acceptor is higher in energy, an increased value of V_oc_ can be reached. The following expression can be used to produce V_oc_ values:

(5) $$V_{oc}=\left(\frac{1}{e}\right)\;\left[\left|E_{HOMO}^{Donor}\right|-\left|E_{LUMO}^{Acceptor}\right|\right]-0.3$$

where “e” represents the electron charge and 0.3 represents the empirical factors [66,67]. The primary goal of V_oc_ is to match the HOMO of the recognized PBDB-T donor with the LUMO of designed compounds. PBDB-T polymer offers a high-performance donor with HOMO and LUMO energy levels of −5.33 eV and −2.92 eV, respectively, based on available resources [68]. Then, we have evaluated the difference between the LUMO energy levels of our proposed compounds and the HOMO energy level of donor polymer (PBDB-T). Figure 8 lists the assembled results, and the descending order of V_oc_ is M4 (1.78 V) > M6 (1.76 V) > M5 (1.69 V) > M1 (1.62 V) > M3 (1.59 V) > M2 (1.54 V). The alignment of the donor (PBDB-T) and the acceptor (M1–M6) energy levels predicts a high open-circuit voltage V_oc_; a significant overlap between the HOMO of the donor and the LUMO of the acceptor suggests a high V_oc_. Organic compounds having a relatively moderate optical band gap have been discovered to have high V_oc_ values, which is consistent with previous studies [69,70,71,72]. Nevertheless, despite their potential, these theoretical forecasts include inherent uncertainties. They should therefore be regarded as first estimates, and experimental validation is necessary to verify the anticipated values. It should be mentioned that bromine substitution combined with positional change of the sulfur atom, leads to a 0.24 V difference in V_oc_ values. It is therefore possible to successfully improve organic solar cells by employing these recently suggested molecules with V_oc_ values exceeding 1.5 V.

Effective dissociation of the exciton requires the electron to move from the LUMO of the donor to the LUMO of the acceptor. As seen in Figure 8, the energy difference between the LUMOs of the PBDB-T and designed compounds denoted LUMO_offset_ is greater than 0.3 eV, which ensures charge transfer in organic photovoltaic devices.

Higher fill factor (FF) and V_oc_ are necessary for higher power conversion efficiency (PCE). The expected values for fill factor are typically larger than they are in reality since shunt and series resistances are ignored. However, it continues to be a significant element influencing the effectiveness of a device’s photovoltaic efficiency. The following equation can be used to compute the fill factor.

(6) $$FF=\frac{\frac{e\;V_{oc}}{K_{B}\;T}-\ln\;(\frac{e\;V_{oc}}{K_{B}\;T}+0.72)}{\frac{e\;V_{oc}}{K_{B}\;T}+1}$$

The normalized V_oc_ is represented by the formula above as (eV_oc_/K_B_T) [73,74]. While K_B_ is the Boltzmann constant, T is the temperature (300 K), and e is the electron charge. As seen in Table 6, M4 and M6 molecules showed the highest fill factor values of 0.925 and 0.924, respectively. This demonstrates the amazing ability of the M6 molecule to efficiently convert solar sunlight into electrical energy, increasing photovoltaic efficiency.

## 4. Conclusions

In summary, the theoretical investigations of the six designed phenothiazine-based novel material compounds, which were classified into two groups based on the thienyl position of the fused-ring electron acceptor, show that bromination significantly affects the optoelectronic properties of these compounds. We have employed density-functional theory (DFT) and time-dependent DFT to explore the impact of structural modifications on the optoelectronic properties of newly designed D-π-A material compounds (M1–M6). With the donor (phenothiazine) and π-bridge (furan) held constant, we assessed the impact of bromine atom substituents and the change of sulfur atom positions on the optoelectronic properties of the tailored compounds. Substituting the bromine atom and repositioning the thienyl group within the fused IC terminal acceptor reduced the energy gap from 2.30 to 2.14 eV due to their planar geometries. We demonstrated that all of the designed compounds showed an impressive ability to absorb energy in the visible and near-IR regions, which is stated from 400 nm to nearly 950 nm. Brominated structures showed a slight red shift in the maximum absorption as compared to non-substituted compounds. Moreover, the energy levels of all proposed compounds align well with those of the PBDB-T polymer donor, resulting in a V_oc_ value between 1.54 V and 1.78 V, which is crucial for achieving optimal performance in photovoltaic devices. Through an extensive analysis of the electron localization function, reactivity parameter values, transition density matrix, reduced density gradient, optical properties, charge transfer length, overlap integral, and HOMO-LUMO energy gap, we have revealed pivotal information about these novel proposed compounds. The results suggest that substituting bromine atoms in our studied molecules improves their efficiency as materials for transporting holes. These results show how combining the change of sulfur atom position of the end-group associated with bromine substitution can significantly enhance the performance of novel material compounds, providing valuable guidance for the design of more efficient organic photovoltaic devices.

## Figures and Tables

**Table 1:** Assessment of planarity of all studied compounds: bond length (L_c-c_), dihedral angle (θ), MPP, and SDP.

Compounds	Bond Length L_c-c_ (Å)	Dideral Angle θ (°)	Molecular Planarity Parameter (Å)	Span of Deviation (Å)
M1	1.406	0.032	0.437	1.980
M2	1.405	0.390	0.454	2.091
M3	1.408	2.253	0.770	2.786
M4	1.410	0.053	0.433	1.970
M5	1.409	0.064	0.435	1.980
M6	1.411	1.458	0.479	2.110

**Table 2:** Chemical reactivity parameters of all studied compounds (M1–M6).

Compound	HOMO (eV)	LUMO (eV)	E_g_ (eV)	η (eV)	μ (eV)	ω (eV)	ρ (Debye)
M1	−5.61	−3.41	2.20	1.10	−4.51	9.25	10.56
M2	−5.63	−3.49	2.14	1.07	−4.56	9.72	10.65
M3	−5.60	−3.44	2.16	1.08	−4.52	9.46	10.90
M4	−5.55	−3.25	2.30	1.15	−4.40	8.42	9.23
M5	−5.58	−3.34	2.24	1.12	−4.46	8.88	9.94
M6	−5.56	−3.27	2.29	1.14	−4.41	8.53	10.25

**Table 3:** Simulated values of optical parameters:λ_max_, energy excitation (E), oscillator strength (f), and electronic transition contributions.

Compounds	λ_max_ (nm)	Energy E (cm^−1^)	Oscillator Strength f	Contribution
M1	638.10	15,671.46	0.41	HOMO → LUMO (98%)
M2	654.02	15,289.95	0.39	HOMO → LUMO (98%)
M3	648.88	15,410.94	0.40	HOMO → LUMO (98%)
M4	620.47	16,116.68	0.25	HOMO → LUMO (96%)
M5	639.12	15,646.45	0.20	HOMO → LUMO (95%)
M6	626.49	15,961.82	0.16	HOMO → LUMO (92%)

**Table 4:** Simulated results of the first excited state for all proposed compounds including the centroid distance (D_index_), electron–hole overlap (S_r_), degree of separation (t), and charge transfer length (∆r).

Compound	D_index_ (Å)	S_r_	t_index_ (Å)	∆r (Å)
M1	6.243	0.456	3.165	6.242
M2	6.282	0.454	3.169	6.281
M3	6.427	0.446	3.356	6.423
M4	7.338	0.370	4.511	7.007
M5	7.507	0.352	4.725	7.143
M6	7.713	0.321	5.074	7.244

**Table 5:** Simulated exciton binding energy for all proposed compounds.

Compound	E_g_ (eV)	E_opt_ (eV)	E_b_ (eV)
M1	2.20	1.94	0.26
M2	2.14	1.90	0.24
M3	2.16	1.91	0.25
M4	2.30	2.00	0.30
M5	2.24	1.94	0.30
M6	2.29	1.98	0.31

**Table 6:** Calculated normalized V_oc_ and fill factor (FF) of all proposed structures.

Compound	V_oc_ (V)	Normalized V_oc_ (V)	FF
M1	1.62	62.69	0.919
M2	1.54	59.59	0.916
M3	1.59	61.53	0.918
M4	1.78	68.88	0.925
M5	1.69	65.40	0.922
M6	1.76	68.11	0.924

## Data Availability

The original contributions presented in this study are included in the article/Supplementary Materials. Further inquiries can be directed to the corresponding author(s).

## Supplementary Materials

The following supporting information can be downloaded at: https://www.mdpi.com/article/10.3390/polym17010115/s1, Figure S1: Optimized molecular structures of EG1 and EG2 and their monobrominated isomers; Figure S2: Electron Localization Function (ELF) map for EG1 and EG2 and their monobrominated structures; Table S1: Calculated HOMO (eV), LUMO (eV), and dipole moment (Debye) of the monobrominated isomers of end groups (EG1 and EG2).
