# Supplementary material for: Exploring the Impact of Structural Modifications of Phenothiazine-Based Novel Compounds for Organic Solar Cells: DFT Investigations
Source: Polymers (Basel). 2025 Jan 5;17(1):115. doi: 10.3390/polym17010115 (PMC11722700; doi:10.3390/polym17010115)
Supplement: Supplementary file 1 [file polymers-17-00115-s001.zip › polymers-3367953-supplementary.pdf]

Supplementary materials

Exploring the Impact of Structural Modifications of Phenothiazine-Based Novel Compounds for Organic Solar Cells: DFT Investigations

Walid Taouali[1,*], Amel Azazi [2,*], Rym Hassani [3], Entesar H. EL-Araby[2] and Kamel Alimi [1]

1 Research Laboratory of Asymmetric Synthesis and Molecular Engineering of Materials for Organic Electronic (LR18ES19), Department of Physics, Faculty of Sciences of Monastir, University of Monastir,
Avenue of Environment, Monastir 5019, Tunisia; kamel.alimi@fsm.rnu.tn
2 Department of Physical Sciences, Physics Division, College of Science, Jazan University,
P.O. Box 114, Jazan 45142, Saudi Arabia; eelaraby@jazanu.edu.sa
3 Environment and Nature Research Centre, Jazan University, P.O. Box 114, Jazan 45142, Saudi Arabia;
rhassani@jazanu.edu.sa
* Correspondence: tawali_walid@yahoo.fr (W.T.); aazazi@jazanu.edu.sa (A.A.)

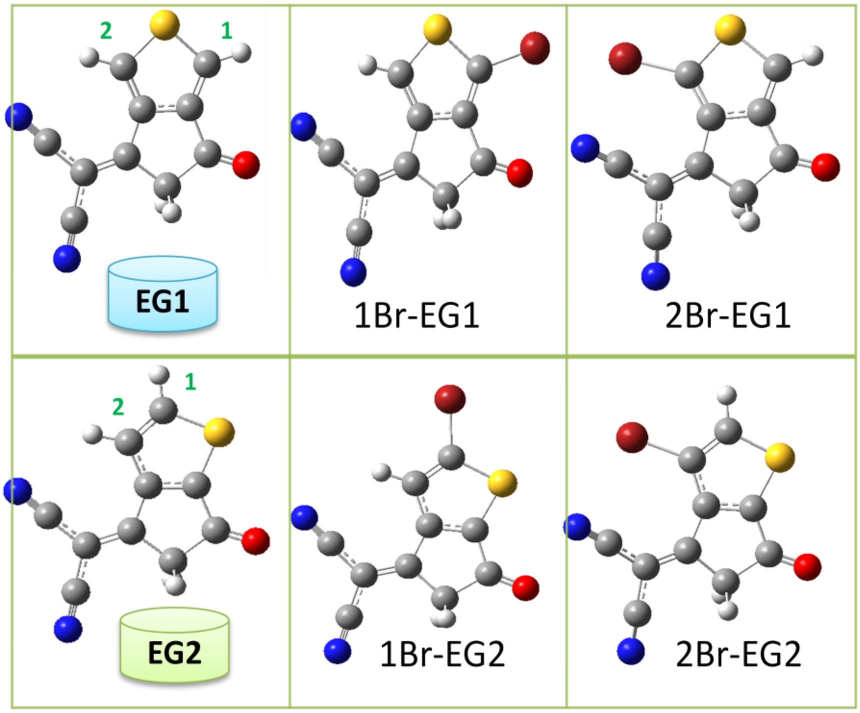

Figure S1: Optimized molecular structures of EG1 and EG2 and their mono-Briminated isomers.

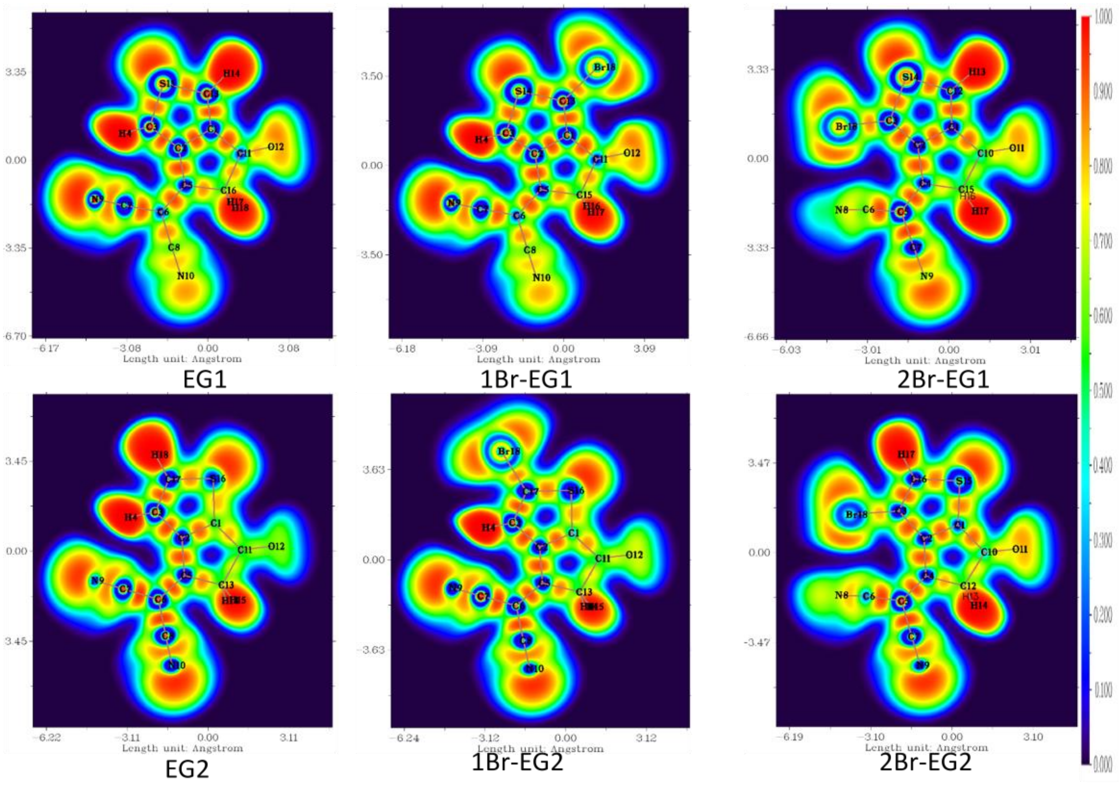

Figure S2: Electron Localization Function (ELF) map for EG1 and EG2 and their mono-Brominated structures.

	**END GROUP 1**			**END GROUP 2**		
	**EG1**	**1Br-EG1**	**2Br-EG1**	**EG2**	**1Br-EG2**	**2Br-EG2**
LUMO	-3.42	-3.52	-3.51	-3.66	-3.79	-3.77
HOMO	-7.62	-7.50	-7.50	-7.65	-7.60	-7.74
Dipole moment	5.05	4.43	5.75	4.91	4.08	5.60

Table S1: Calculated HOMO (eV), LUMO (eV) and dipole moment (Debye) of the mono-brominated isomers of end-groups (EG1 and EG2).
